# Evaluation of a Thiolated Chitosan Scaffold for Local Delivery of BMP-2 for Osteogenic Differentiation and Ectopic Bone Formation

**DOI:** 10.1155/2013/878930

**Published:** 2013-08-20

**Authors:** In-Ho Bae, Byung-Chul Jeong, Min-Suk Kook, Sun-Hun Kim, Jeong-Tae Koh

**Affiliations:** ^1^Department of Pharmacology and Dental Therapeutics, School of Dentistry, Chonnam National University, Gwangju 500-757, Republic of Korea; ^2^Research Center for Biomineralization Disorders, School of Dentistry, Chonnam National University, Gwangju 500-757, Republic of Korea; ^3^Dental Science Research Institute, School of Dentistry, Chonnam National University, Gwangju, 500-757, Republic of Korea

## Abstract

Thiolated chitosan (Thio-CS) is a well-established pharmaceutical excipient for drug delivery. However, its use as a scaffold for bone formation has not been investigated. The aim of this study was to evaluate the potential of Thio-CS in bone morphogenetic protein-2 (BMP-2) delivery and bone formation. *In vitro* study showed that BMP-2 interacted with the Thio-CS and did not affect the swelling behavior. The release kinetics of BMP-2 from the Thio-CS was slightly delayed (70%) within 7 days compared with that from collagen gel (Col-gel, 85%), which is widely used in BMP-2 delivery. The BMP-2 released from Thio-CS increased osteoblastic cell differentiation but did not show any cytotoxicity until 21 days. Analysis of the *in vivo* ectopic bone formation at 4 weeks of posttransplantation showed that use of Thio-CS for BMP-2 delivery induced more bone formation to a greater extent (1.8 fold) than that of Col-gel. However, bone mineral density in both bones was equivalent, regardless of Thio-CS or Col-gel carrier. Taken together, Thio-CS system might be useful for delivering osteogenic protein BMP-2 and present a promising bone regeneration strategy.

## 1. Introduction

Current approaches for bone regeneration such as autografts and allografts face significant limitations [1]. Various factors including limited supply, risk of immune rejection, and chronic immune responses have prompted interest in bone graft substitutes. Many growth factors for bone formation have been reported. Bone morphogenetic protein-2 (BMP-2) is generally acknowledged due to its superior activity. It has been used in dental and orthopedic biomaterials to promote bone formation because of its strong osteogenic activity. BMP-2 induces bone formation *in vivo* [2–7], presumably by stimulating mesenchymal stem cell differentiation into an osteoblast lineage and by increasing the number of differentiated osteoblasts capable of forming bone [8]. This stimulatory effect of BMP-2 on osteoblastic differentiation is of major importance during bone healing. Despite its strong osteoinductive activity, the systemic delivery of BMP-2 can be impractical and undesirable because it may have uncontrolled adverse effects, such as unwanted ectopic bone formation. In addition, clinical use of BMP-2 has been limited by the lack of suitable delivery systems. Systems evaluated as carriers to localize BMP-2 include porous hydroxyapatite (HA) [9], absorbable collagen [10], polylactic acid [11], polylactic-co-glycolic acid [12], demineralized bone powder, and bovine collagen type sponges [13]. Although HA is a biocompatible material, it is not biodegradable. Therefore, it remains at the defect site. Collagen gel (Col-gel) can be immunogenic, and demineralized bone powder suffers from insufficient supply and poor characterization as a delivery system. Thus, an efficacious delivery system (i.e., scaffold) is still required to localize BMP-2 at the desired site. Natural biomaterials are widely used for scaffold fabrication in tissue engineering because they facilitate cell attachment and maintenance of the differentiation function. Chitosan (CS), obtained by alkaline deacetylation of chitin, is one of the most abundant polysaccharides in nature. It has received considerable attention in a variety of areas such as pharmaceutics [14], tissue engineering [15], antimicrobial agents [16], and chromatography [17] because of its properties, which include enzymatic biodegradability, nontoxicity, and biocompatibility, even when used in human and animal models [18–20]. However, CS suffers from limited solubility at physiological pH and causes presystemic metabolism of drugs in the presence of proteolytic enzymes [21]. These inherent drawbacks of CS have been overcome by forming derivatives such as carboxylated CS [22], adding various conjugates [23], thiolated CS [24] or acylated CS [25]. Among these various CS derivates, thiomer technology has a range of advantages for drug delivery such as sustained drug release [26] and high stability [24]. The usefulness of thiolated chitosan (Thio-CS) as a scaffold for controlled drug release has been demonstrated by means of model drugs such as clotrimazole [27], salmon calcitonin [28], insulin [29], and tobramycin [30]. However, most of the research has focused on systemic drug delivery such as neural tissue [31], peroral peptide delivery [32], and nasal administration [33]. Despite the advantages of Thio-CS for tissue engineering, the potential application of this material for bone tissue has not been investigated. The aim of this study was to evaluate the physicochemical properties of Thio-CS for BMP-2 delivery and bone formation *in vitro *and *in vivo*.

## 2. Materials and Methods

### 2.1. Fabrication of Thio-CS

To obtain a 1% (w/v) solution, 500 mg of CS (average molecular mass: 400 kDa, Fluka GmbH, Buchs, Switzerland) was dissolved in 50 mL of 1% acetic acid by stirring the mixture for 1 h. Traut's reagent (2-iminothiolane-HCl, 2-IT) was used for the immobilization of thiol groups to primary amino groups of proteins and the modification of CS. We have previously reported the optimal conditions for fabricating Thio-CS [34]. In brief, the pH of a mixture containing a 1% solution of CS and 0.1 mg/mL of 2-IT was adjusted, ranging from 4 to 12. The mixtures were then incubated for 30 min at room temperature. To investigate the time effect of the air oxidation on disulfide bond formation, the samples were incubated for 3-day intervals at pH 7 under stirring. To remove unreacted agent, the resulting mixture was dialyzed with several exchanges of the dialyzing solution. To prevent more oxidation of samples, 5 mM or 0.4 mM of HCl solution was used as a dialyzing solution depending on dialysis step. The samples were freeze-dried at −80°C and 0.01 mbar (Christ Beta 1–8 K; Germany) and stored at 4°C until further use.

### 2.2. Determination of the Thiol Group and Disulfide Bond

Ellman's reagent (3 mg of 5,5′-dithiobis (2-nitrobenzoic acid)) (Sigma, St. Louis, MO, USA) was used to quantify the amount of thiol groups on the modified CS, as described previously [34]. Briefly, 5 mg of the freeze-dried samples was dissolved in 2.5 mL of demineralized water. Then, 250 *μ*L of the samples, 250 *μ*L of 5 M phosphate-buffered saline (PBS, pH 8.0), and 500 *μ*L of Ellman's reagent dissolved in 10 mL of 0.5 M PBS (pH 8.0) were reacted in the same tube. The reaction was allowed to proceed for 2 h at room temperature. After removal of the precipitated polymer by centrifugation (24,000 ×g; 5 min), 300 *μ*L of the supernatant was transferred to a microtitration plate, and the absorbance was immediately measured at 450 nm (Bio-Tek Instruments, Winooski, VT, USA). The amount of thiol moieties was calculated from a standard curve of absorbance obtained from solutions with increasing concentrations of L-cysteine hydrochloride hydrate (Sigma-Aldrich, Steinheim, Germany). Disulfide bond content within precipitate or reacting solution was determined after reduction with NaBH_4_ and addition of Ellman's reagent as described by Habeeb [35]. The degree of cross-linking of such materials is usually determined by a chemical analysis method using 2,4,6-trinitrobenzenesulfonic acid (TNBS), labeling of residual amine groups [36]. To 0.3 mL of sample, 0.3 mL of NaHCO_3_ (4%) and 0.3 mL of TNBS (0.1%) were added. The solution was allowed to react for 2 h at 40°C, and then 0.3 mL of sodium dodecyl sulfate (10%) and finally 0.17 mL HCl (1 M) were added. The absorbance of the resulting solution was read photometrically at 335 nm, against a blank but with 0.3 mL of H_2_O instead of the sample.

### 2.3. Evaluation of the Swelling Behavior

To understand the effect of the molecular transport of liquids into Thio-CS, the water-absorbing (i.e., swelling) capacity was determined by gravimetric methods. To measure the weight of Thio-CS after swelling, 0.1 g of Thio-CS was placed in trans-well (Corning Inc., Corning, NY, USA). The trans-well was then placed into a 24-well culture dish containing a physiological solution of 1 mL of PBS (pH 7.0) and incubated at room temperature. The swelling ratio was measured by comparing the change in the weight of Thio-CS before and after incubating. The percentage of swelling ratio was calculated by the following formula:

(1) $$\begin{array}{c} \text{Swelling}\;\;\text{ratio}\;\left(\%\right)=\frac{\left(W_{w}-W_{i}\right)}{W_{i}}\times 100\%, \end{array}$$

where *W*_*w*_ is the weight of the swollen Thio-CS and *W*_*i*_ is the initial weight of the Thio-CS.

### 2.4. Scanning Electron Microscopy

The morphologies of the samples were examined using scanning electron microscope (SEM) (Hitachi, Tokyo, Japan). As moisturized materials cannot be detected by SEM, the samples were lyophilized. Prior to imaging, the samples were fixed and dehydrated. The Thio-CS was soaked in a primary fixative of 2.5% glutaraldehyde (Sigma) for 2 h. The samples were dehydrated by replacing the buffer with increasing concentrations of ethanol (from 40 to 100%) for 10 min each. They were then dried at room temperature for 24 h and subjected to SEM at voltages ranging from 5 to 15 kV after the samples were sputter coated in white gold.

### 2.5. Delivery of BMP-2 Using Thio-CS

 The freeze-dried and sponge-shaped Thio-CS was placed in 0.1 mg/mL of BMP-2 (R&D Systems, Minneapolis, MN, USA) solution. The mixture of Thio-CS and BMP-2 was gelated within a few of minute at the room temperature, and we designated it as Thio-CS-B2. Type I collagen gel (Col-gel), which is widely used as a drug delivery system, was employed as a control for BMP-2 delivery. Col-gel was prepared from acid solubilized type I collagen stock solution, which is extracted from rat tail tendon (BD Biosciences, San Jose, CA, USA). According to the recommendation of manufacturer, the stock solution was adjusted to final 3 mg/mL of collagen solution containing 0.1 mg/mL of BMP-2, and it was gelated at 37°C (Col-gel-B2). For the evaluation of BMP-2 kinetics, Thio-CS-B2 or Col-gel-B2 was placed in trans-well, and subsequently the trans-well was assembled with 24-well plate filling with alpha-minimum essential medium (*α*-MEM, Gibco, Gaithersburg, MD, USA). Both gels were incubated for the designated time at 4°C while shaking gently. The amount of BMP-2 in medium was measured by using the BMP-2 enzyme-linked Immuno-sorbent assay kit (Invitrogen, Carlsbad, CA, USA). 

### 2.6. Cell Culture and Proliferation Assay

Preosteoblast MC3T3-E1 cells (1 × 10^4^ cells/cm^2^) were cultured in *α*-MEM, containing the BMP-2 released from scaffolds as described previously, 10% fetal bovine serum (FBS), 100 U/mL of penicillin, and 100 *μ*g/mL of streptomycin (Gibco) in humidified air containing 5% carbon dioxide at 37°C. In case of inducing osteoblast differentiation, 50 *μ*g/mL of ascorbic acid and 5 mM of *β*-glycerophosphate were added, and the culture media was changed every 3 days. To investigate the cytotoxicity of the Thio-CS on MC3T3-E1, the XTT assay was performed by using an EZ-cytox cell viability assay kit (Daeil lab service Co., Seoul, Republic of Korea) for 1, 4, 7, 14, and 21 days. Briefly, 10 *μ*L of EZ-cytox reagent was added to the cell culture dish. By the action of mitochondrial dehydrogenases, XTT was metabolized to form a formazan dye, which was spectrophotometrically determined by measuring the absorbance at 450 nm using a microplate reader (Bio-Tek Instruments, Winooski, VT, USA). The amount of formazan salt formed corresponds to the number of viable cells contained in each well.

### 2.7. Sodium Dodecyl Sulfate Polyacrylamide Gel Electrophoresis and Western Blotting

The structural integrity of BMP-2 in the Thio-CS was detected by sodium dodecyl sulfate polyacrylamide gel electrophoresis (SDS-PAGE). In brief, BMP-2 containing Thio-CS was degraded by chitosanase (10 mU/mL) for 1 h at 37°C. The samples were then mixed with the loading buffer without a reducing agent. The SDS-PAGE was performed with 15% separating gel at a constant voltage mode (100 V). Finally, the gel was stained with 1% Coomassie brilliant blue solution and was destained with an aqueous solution of 10% methanol and 10% acetic acid. To detect the BMP-2, Western blot analysis was performed. The samples were underwent 15% SDS-PAGE and were electrotransferred onto polyvinylidene fluoride membranes. The blots were blocked with a buffer containing 0.05% Tween-20 and 5% skimmed milk and reacted sequentially with primary and secondary antibodies. The primary antibody against BMP-2 (Santa Cruz Biotechnology) and horseradish peroxidase-conjugated secondary antibodies (KPL, Gaithersburg, MD, USA) were used at 1 : 1,000 and 1 : 2,000 dilution, respectively. The antigen-antibody complexes were visualized using the enhanced chemiluminescence image analyzer LAS 4000 mini (Fuji Film, Tokyo, Japan).

### 2.8. Measurement of Alkaline-Phosphatase Activity and Calcium Mineral Deposition

The MC3T3-E1 cells were cultured for 7, 14, and 21 days as described above. The cells were lysed, and the lysates were then used to measure the alkalinephosphatase (ALP) activity at 1, 2, and 3 weeks. In brief, the cell homogenates reacted with the ALP assay mixtures containing 0.1 M 2-amino-2-methyl-1-propanol (Sigma, St. Louis, MO, USA), 1 mM MgCl_2_, and 8 mM *p*-nitrophenyl phosphate disodium. After 10 min incubation at 37°C, the reaction was stopped with 0.1 N NaOH, and the absorbance of the resulting solution was measured photometrically at 405 nm. Quantitative double-stranded DNA in the solution was measured using a picogreen dsDNA quantification kit (Molecular Probes, Inc., Eugene, OR, USA) as instructed by the manufacturer. To measure the level of calcium mineral deposition, alizarin-red staining (AR-S) was performed. After 3 weeks in culture, the cells were fixed with 70% ethanol, rinsed five times with deionized water, treated for 10 min with 40 mM of AR-S solution at pH 4.2, and then washed with 1 ×PBS for 15 min with gentle agitation. Stained samples were photographed, followed by a quantitative eluting procedure using 10% (w/v) cetylpyridinium chloride in 10 mM sodium phosphate (pH 7.0) for 15 min at room temperature. The AR-S concentration was determined by comparing it to an AR-S standard curve with an optical density of 540 nm.

### 2.9. Animal Preparation

The ethics committee of Chonnam National University approved the animal study for this research. The effects of the Thio-CS scaffold on ectopic bone formation induced by BMP-2 were studied in mice (C57/BL6, 8 weeks of age, obtained from Damool Science, Daejeon, Republic of Korea). Before transplantation, the mice were anesthetized with a mixture of rumpun (20 mg/kg) and ketamine hydrochloride (20 mg/kg) intramuscularly. The area of transplantation at the dorsum of the mice was shaved and disinfected. Either a Thio-CS-B2 or a Col-gel-B2 scaffold was subcutaneously transplanted into the dorsum of the mice. Transplantations of Col-gel and Thio-CS scaffolds without BMP-2 were used as controls. After transplantation of the scaffolds (*n* = 6 per group), the mice were given access to food and water. Six weeks after injection, the animals were sacrificed by intracardiac injection of KCl, and the implants were isolated and fixed in 10% formaldehyde solution for subsequent analysis. 

### 2.10. Evaluation of Ectopic Bone Formation

Isolated samples were subjected to microcomputed tomography (microCT; Skyscan, Kontich, Belgium) and histological analysis. The bone volume (BV) and the bone mineral density (BMD) of the isolated implants were determined by using microCT in the cone-beam acquisition mode. The X-ray source was set at 50 kV and 200 *μ*A with a pixel size at 17.09 *μ*m. The exposure time was 1.2 sec. Four hundred fifty projections were acquired over an angular range of 180° (angular step of 0.4°). The tomographic acquired images were transformed into sliced volumetric reconstruction using the Nrecon program (Skyscan) and analyzed using 3D CT analyzer software (CTAN, Skyscan). The BMD of the isolated samples was calibrated from Hounsfield units (HU) of 0.25 and 0.75 mg/cm^3^ of the hydroxyapatite density phantom. For histological analysis, isolated specimens were serially sliced, decalcified in 8% formic acid, and embedded in paraffin wax. Five micrometer-thick sections were stained with hematoxylin and eosin (H&E) for histological assessment. 

### 2.11. Statistical Analysis

The difference between the Thio-CS and the Col-gel in the release kinetics of BMP-2 was statistically compared by using the ANOVA test. The statistical differences in biocompatibility, ALP activity, and AR-S were analyzed by the Student's *t*-test. The results are expressed as the mean ± standard deviation (SD) from three or more separate experiments. A value of *P < 0.05, **P < 0.005 was considered statistically significant.

## 3. Results and Discussion

### 3.1. Fabrication of Thio-CS and Physicochemical Properties

**Figure 1:**
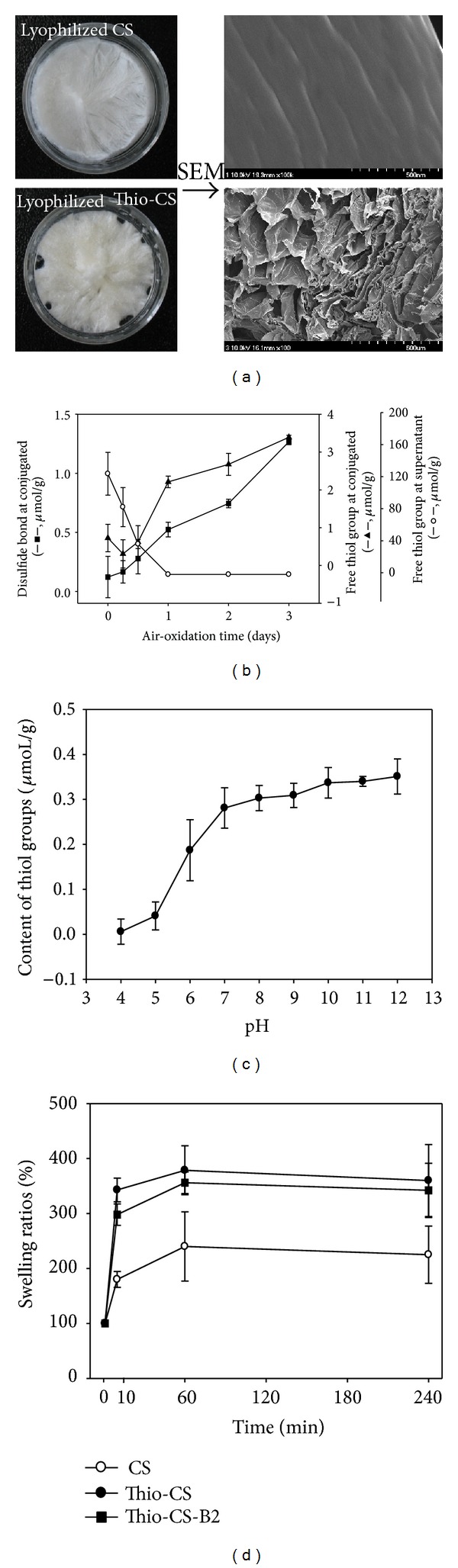
Physicochemical properties of Thio-CS. (a) Photograph of lyophilized CS and Thio-CS (left panel) and its SEM morphologies (right panel, 100x), (b) determination of free thiol or disulfide bond according to the air-oxidation time, (c) influence of the pH on the formation of disulfide bonds, and (d) effect of Thio-CS containing BMP-2 on swelling. The amount of the thiol groups in the supernatant or the pellet after centrifugation was determined spectrophotomertically using Ellmans's reagent to quantify the free thiol groups, as described in Section 2. The values indicated are means ± SD (*n* = 3).

The reagent 2-IT has been widely used for the immobilization of thiol groups to primary amino groups of proteins [37]. Since moisturized materials cannot be detected by SEM, unmodified CS and Thio-CS were lyophilized. As shown in Figure 1(a), SEM images of Thio-CS showed honeycomb-like pores structure. It assumed that the pore of Thio-CS occurred by increasing molecular weight of CS through disulfide bonding each other. This is consistent with previous report that pore size of substance has direct relationship with the molecular weight of materials [38]. As we reported previously, the optimum ratio between 2-IT and CS for the formation of disulfide bonds is 0.1 mg/mL and 1% (w/v) [34]. However, this ratio does not take account of the air-oxidation time, which can affect the formation of disulfide bonds between CS polymers. Therefore, in this study, we investigated the variation of the disulfide bond and the free sulfate at the conjugate or the supernatant, according to the air-oxidation time. As shown in Figure 1(b), the free thiol content in the supernatant was decreased with the air-oxidation time. However, the free thiol and disulfide groups in the conjugate were increased. Moreover, the amount of residual amino groups in CS was decreased in the Trinitrobenzene sulfonate assay (data not shown). These results suggest that the thiol moiety of 2-IT was transferred to the amino group of CS, that it can be formed by cross-linking between CS polymers, and that these phenomena depend on the air-oxidation time. The cross-linking of the polymeric chains such as inter- or intramolecular disulfide bonds might result in high stability for drug delivery systems. Moreover, the formation of the disulfide bond was increased with pH and was saturated at pH 7 (Figure 1(c)). The swelling property of the scaffold plays crucial roles in cell growth, cell adhesion, nutrient perfusion, and tissue regeneration [39]. Many researchers have attempted to measure the swelling ratio of materials using the gravimetric method [40]. As the purpose of this study was to evaluate the potential use of Thio-CS for BMP-2 delivery, the swelling property of Thio-CS was compared with that of Thio-CS-B2, which contained BMP-2. Unmodified CS was used as a negative control. The weight of the Thio-CS increased significantly, up to 3.5 times within 10 min compared with its initial weight, and this was maintained continuously. A similar phenomenon was observed with Thio-CS-B2, although the rate was slightly lower than that of the Thio-CS. However, the weight of unmodified CS was lower (approximately 65% at 60 min) than that of the others (Figure 1(d)). These results suggest that BMP-2 does not affect the swelling property Thio-CS.

### 3.2. Interaction between Thio-CS and BMP-2

**Figure 2:**
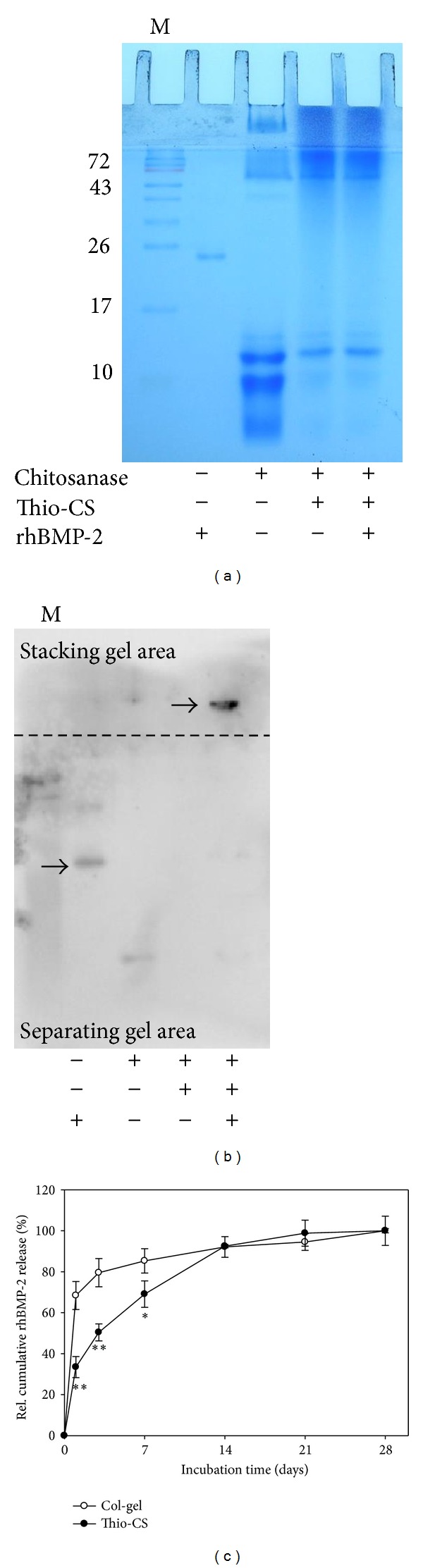
Evaluation of the interaction between BMP-2 and Thio-CS. The interaction was evaluated by SDS-PAGE (a) and Western blotting (b). (c) *In vitro* cumulative release of BMP-2 from the Thio-CS and the Col-gel. The arrow indicates BMP-2 which detected by the BMP-2 antibody. M is a molecular weight marker. Black dotted line is the border line between the stacking and the separating gel. The indicated values are means ± SD (*n* = 3), *P < 0.05, **P < 0.005 as compared with those of the Col-gel at the same time point.

As the swelling property is insufficient to explain the interaction between the Thio-CS and BMP-2, the structural integrity was investigated. Thio-CS-B2 was incubated with chitosanase, and the reactant was subjected to SDS-PAGE and Western blotting. It was difficult to assay Thio-CS without chitosanase because its viscosity was too high to subject to SDS-PAGE (data not shown). The results pointed to several bands in the chitosanase and the Thio-CS groups (Figure 2(a)). Basically, Thio-CS is a polysaccharide polymer, but it may contain other impurities. However, BMP-2-like molecules were not observed in the Thio-CS. This was confirmed by Western blotting, which detected the specific BMP-2 antibody (Figure 2(b)), indicating that Thio-CS does not contain BMP-2-like molecules. The Western blot analysis showed that the BMP-2 signal was observed only in the BMP-2 and the Thio-CS-B2 group. The size of the control BMP-2 peptide that was detected was approximately 25 kDa. However, the signal at the lane of Thio-CS-B2 was detected in the stacking gel area, indicating that it was not mobilized. In other words, if there was not any interaction between BMP-2 and Thio-CS, BMP-2 should be mobilized to the separating gel area. These results suggested that BMP-2 interacted with Thio-CS, although the mechanism is unknown, and that BMP-2 was located in Thio-CS. It is likely that this interaction between BMP-2 and Thio-CS may affect delaying the release of BMP-2 from Thio-CS rather than simply absorbing.

### 3.3. **In Vitro ** Release of BMP-2

Based on previous physicochemical properties of Thio-CS (Figure 1 and [34]), it is expected to delay the velocity of the release of BMP-2. Therefore, the release kinetics of BMP-2 from Thio-CS were measured for 28 days and compared with those of the Col-gel, which is widely used in the delivery of BMP-2 (Figure 2(c)). As expected, the release velocity of BMP-2 delayed in the Thio-CS group (70% within 7 days) compared with that of the Col-gel (85% within 7 days). The cumulative BMP-2 released from the Thio-CS and the Col-gel almost reached a plateau at 21 and 14 days, respectively. These results suggest that the release velocity of the Thio-CS group showed a sustained pattern of release compared with that of the Col-gel.

### 3.4. Biocompatibility of Thio-CS

**Figure 3:**
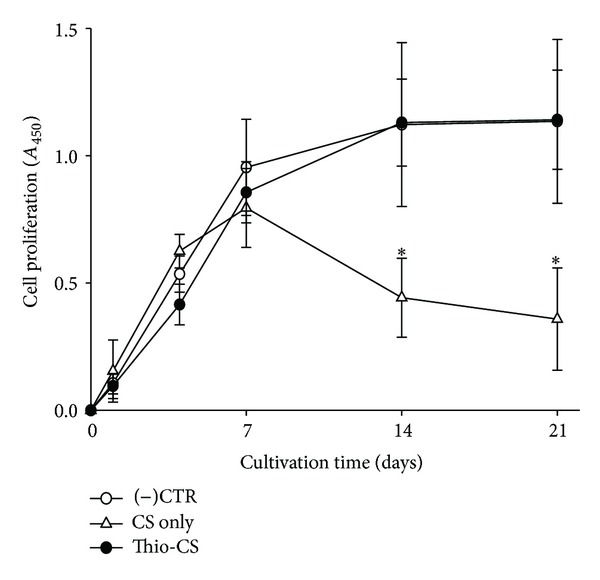
Biocompatibility of Thio-CS by XTT assay. The no treatment (i.e., cell only) group was used as a control. The values indicated are means ± SD, (*n* = 3). *P < 0.05 as compared with that of the control at the same time point.

Biocompatibility of Thio-CS or CS was evaluated with an XTT assay. The assay system was based on the absorbance of cell lysate and widely used to measure cell viability or proliferation because it relies on the reagent binding only to the mitochondria of living cells.  Figure 3 shows viability of the osteoblast cells, which were cultured with Thio-CS or CS gel for 21 days. No treated cells were used as a control. Cell population in the control was continuously increased with the cultivation time. And pattern of cell proliferation in Thio-CS group was similar to that in the control. However, cell proliferation in CS-treated group was decreased after 14 days. The decreases seem to be related to the gelation or degradation property of CS gel, because the CS gel was more soluble or fragile in the culture medium than Thio-CS gel. Although CS gel itself negatively affected cell viability, Thio-CS gel did not. Based on the results, *in vivo* study progressed with Thio-CS. 

### 3.5. Induction of Osteoblast Differentiation with BMP-2 Delivery Using Thio-CS

**Figure 4:**
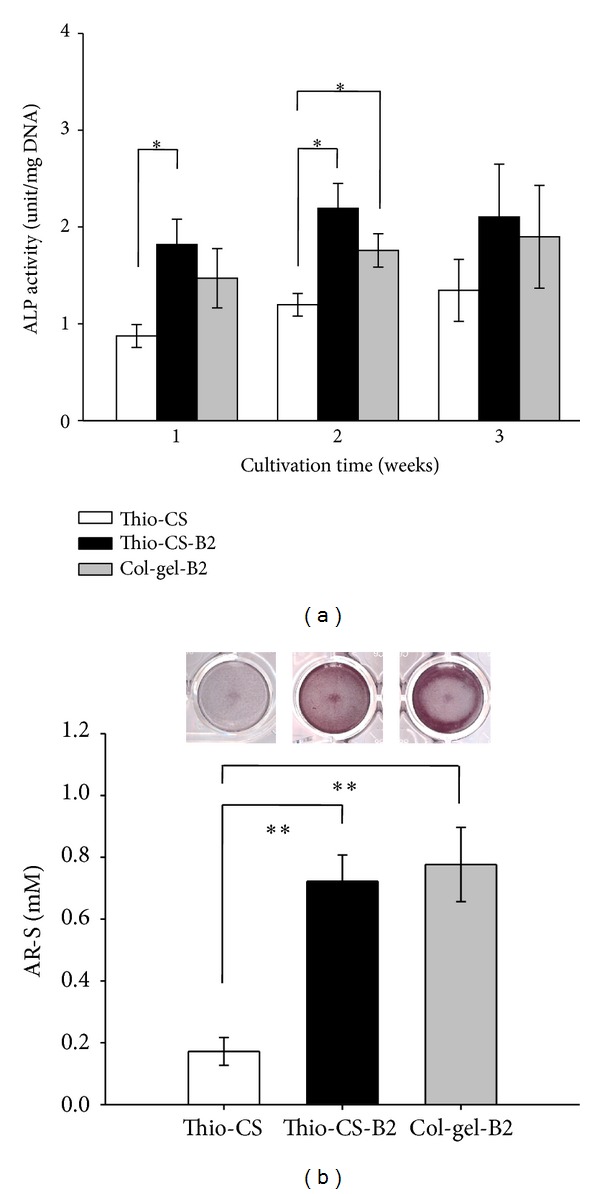
Effects of Thio-CS-B2 on *in vitro* ALP activity (a) and the level of calcium mineral deposition (b). The level of calcium deposition in 3-week culture was evaluated by AR-S. The values indicated are means ± SD, (*n* = 3) *P < 0.05, **P < 0.005 as compared with that of Thio-CS.

The ALP activity and the level of calcium deposition are important considerations for evaluating osteoblast differentiation. ALP activity, which is widely used as a marker for early differentiation of osteoblastic cells and generally expressed before mineralization [41], was measured after 1, 2, and 3 weeks in the MC3T3-E1 cell culture with Thio-CS-B2. The Thio-CS and the Col-gel-B2 were used as controls. As shown in Figure 4(a), the ALP activity was significantly increased in the cells cultured with the Thio-CS-B2 and the Col-gel-B2 compared with that of Thio-CS only. These results suggest that BMP-2 in Thio-CS still retains its biological ability to enhance ALP activity. Calcium mineral deposition is a marker of late differentiation of osteoblastic cells [42]. The level of calcium mineral deposition after 3 weeks in culture was investigated by AR-S. The results showed that calcium deposition in Thio-CS-B2 treated group was increased 4.2 fold compared with that of Thio-CS (Figure 4(b)). Thus, BMP-2, which delivered into the Thio-CS, has a stimulatory effect on the differentiation of osteoblastic cells and on matrix mineralization.

### 3.6. Induction of **In Vivo ** Ectopic Bone Formation by Thio-CS-B2

**Figure 5:**
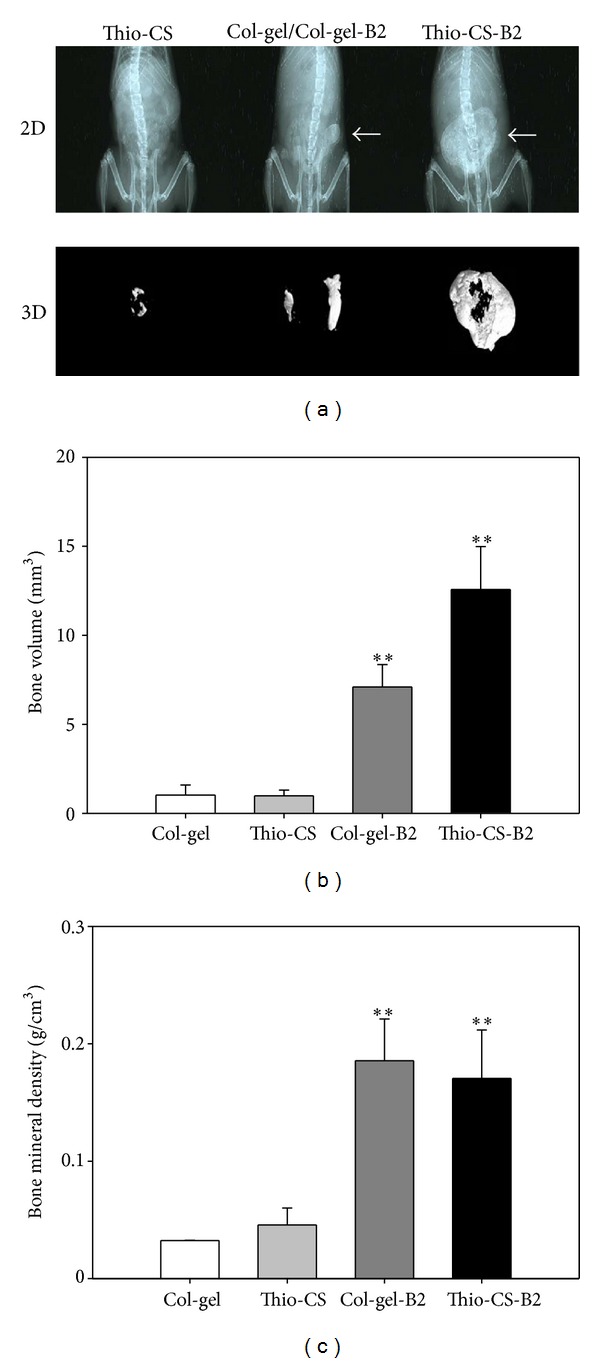
Ectopic bone formation with Thio-CS-B2. Ectopic bone formation was visualized by 2D soft X-Ray and 3D microCT analysis (a). Bone volume (BV) (b) and bone mineral density (c) of the isolated ectopic bones were analyzed by Nrecon and CT analyzer software of Skyscan. The arrows indicate newly formed bone. The values indicated are means ± SD (*n* = 3), **P < 0.005 as compared with control.

**Figure 6:**
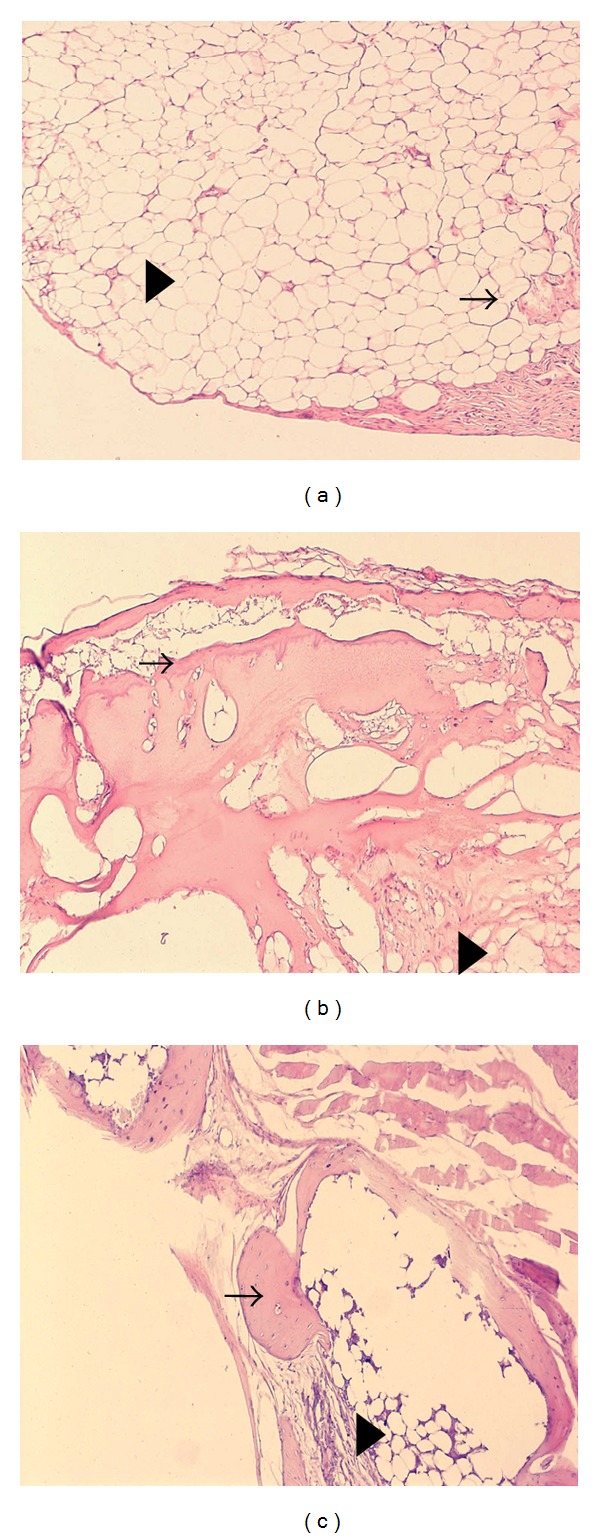
Representative histological sections with H&E staining after 4-week transplantation. (a) Thio-CS only, (b) Col-gel-B2, and (c) Thio-CS-B2. Original magnification is 20x. The arrows indicate newly formed bone tissue, and the triangles indicate residual scaffolds.

BMP-2 is well known to produce ectopic bone formation. This study also determined whether Thio-CS-B2 produces ectopic bone *in vivo* in 8-week-old C57/BL6 mice. Col-gel containing BMP-2 was used as a comparison group. When Thio-CS-B2 was separately implanted in left and right sides of the dorsum in mice, newly formed ectopic bones were observed in both implant sites after 6 weeks. The new bones in Thio-CS-B2 group were big, and each side bone was fused to one. Total bone volume in Thio-CS-B2 group was higher (1.8 fold) than that in the Col-gel-B2 (Figures 5(a) and 5(b)). The enhanced bone formation in Thio-CS-B2 might result from more superior property of Thio-CS than Col-gel for bone formation. In our previous report [34], Col-gel showed the limited swelling property with scant physical change of the porosity, suggesting limiting the capacity to absorb BMP-2. The present study also showed that Col-gel-B2 has the burst release pattern of BMP-2 compared with Thio-CS-B2. On the other hand, Thio-CS produced the sustained release of BMP-2 due to interaction between the gel and the protein (Figure 2). These findings represent that Thio-CS has some advantage for bone formation compared to Col-gel in that Thio-CS could absorb and maintain a greater amount of BMP-2. However, the bone mineral density of the ectopic bone formed by Thio-CS-B2 was not significantly different from that by the Col-gel-B2 (Figure 5(c)). The result suggests that bone quality between both groups would not be different. To yield more accurate observation for bone formation, histological analysis was performed with H&E stain. There was less ectopic bone formation in the Thio-CS group (i.e., without BMP-2, Figure 6(a)). However, new bone with residual scaffolds was observed in Col-gel-B2 and Thio-CS-B2 group (Figures 6(b) and 6(c)).

## 4. Conclusions

In this study, we developed Thio-CS scaffold for BMP-2 delivery and bone formation. The Thio-CS was made by the modification of CS with 2-IT. The 2-IT contributed to *in situ* gel formation of CS via disulfide bonding between the 2-IT-derived thiol groups of the CS polymers, and this disulfide bonding was affected by the air-oxidation time and the pH. The degree of swelling was not affected by BMP-2 addition. Moreover, SDS-PAGE and Western blotting analysis revealed an interaction between BMP-2 and Thio-CS. This interaction may contribute to delaying the release of BMP-2 from Thio-CS. Due to the aforementioned properties of Thio-CS, the release velocity of BMP-2 from Thio-CS was slightly delayed compared with that of the Col-gel. The BMP-2 released from Thio-CS induced osteoblastic differentiation of MC3T3-E1. And the activity of ALP and the level of calcium mineral deposition were also increased. The Thio-CS study did not show any cytotoxicity *in vitro* in XTT assay studies in MC3T3-E1 osteoblastic cells. Based on our results, *in vivo* bone formation studies were performed, and the results showed that BMP-2 containing Thio-CS induced ectopic bone formation to a much greater extent than either the BMP-2 containing Col-gel or the control (no BMP-2). Collectively, these results suggest that the Thio-CS delivery system might be useful for delivering osteogenic protein BMP-2 as a biocompatible synthetic polymer and that it may represent a promising application in bone regeneration strategies.
